# Exploring the correlation and causation between alpha oscillations and one-second time perception through EEG and tACS

**DOI:** 10.1038/s41598-024-57715-6

**Published:** 2024-04-05

**Authors:** Ehsan Mokhtarinejad, Mahgol Tavakoli, Amir Hossein Ghaderi

**Affiliations:** 1 Department of Psychology, Faculty of Education and Psychology, University of Isfahan, Isfahan, Iran; 2 Center for Affective Neuroscience, Development, Learning and Education, University of Southern California (USC), Los Angeles, USA

**Keywords:** Neuroscience, Psychology

## Abstract

Alpha oscillations have been implicated in time perception, yet a consensus on their precise role remains elusive. This study directly investigates this relationship by examining the impact of alpha oscillations on time perception. Resting-state EEG recordings were used to extract peak alpha frequency (PAF) and peak alpha power (PAP) characteristics. Participants then performed a time generalization task under transcranial alternating current stimulation (tACS) at frequencies of PAF−2, PAF, and PAF+2, as well as a sham condition. Results revealed a significant correlation between PAP and accuracy, and between PAF and precision of one-second time perception in the sham condition. This suggests that alpha oscillations may influence one-second time perception by modulating their frequency and power. Interestingly, these correlations weakened with real tACS stimulations, particularly at higher frequencies. A second analysis aimed to establish a causal relationship between alpha peak modulation by tACS and time perception using repeated measures ANOVAs, but no significant effect was observed. Results were interpreted according to the state-dependent networks and internal clock model.

## Introduction

From commenting on the duration of a phenomenon to judging which phenomena lasted longer, time perception plays a crucial role in living and surviving in a variable environment^1–3^. Although humans internally represent time, the neural origin of time perception in the brain, unlike the senses, remains an unsolved problem in cognitive science^4^. Time is a property everywhere, from sight to sound and from pain and happiness to fear. However, time is perceived regardless of specific senses and vastly differs from other perceptions in several respects. First, the lack of specific temporal stimulus in the environment led to the lack of evolutionary specific receptors in the peripheral nervous system to receive a sense of time (e.g., compared to auditory stimuli in the environment and auditory receptors in the ear)^5^. Additionally, the brain lacks a localized dedicated region for time perception (e.g., in comparison to the visual cortex for visual perception), and studies have revealed that time perception recruits many cortical and subcortical regions^6^. Furthermore, disentangling the role of cognitive functions, such as attention, memory, and decision-making, from “pure time perception” remains challenging^3^.

To date, different models have been introduced to explain the neural mechanisms underlying time perception, which can be generally divided into two categories. First, dedicated or centralized approaches^7,8^ usually consider an internal clock (composed of a pacemaker and an accumulator)^9–12^. Second, intrinsic models that suggest whole brain activity may be involved in our sense of time^8,13^. Both categories have found pros and cons to justify time perception in various situations. Despite the existence of models, recent evidence has increasingly pointed towards brain oscillations as promising explanations for the neural mechanisms involved in time perception. Dedicated models view brain oscillations as the “clock ticks” of an internal pacemaker responsible for timekeeping^14^. On the other hand, intrinsic models associate brain oscillations with the activity of state-dependent networks, representing perceived time, or the speed and level of neural information processing and entropy, acting as indicators of perceived time^15,16^. Among neural oscillators, the occipital alpha waves have attracted particular attention^17–24^.

The alpha waves are the most dominant oscillations in the human brain, which have been hypothesized to underlie phasic transmission from the lateral geniculate nucleus (LGN) to the striate and extrastriate cortex. In practice, alpha oscillation is prominent over the posterior area of the brain, particularly in eyes-closed resting state EEG recordings^25^. Within the alpha frequency range, a particular frequency exhibits the greatest power, often resembling a peak. This frequency is referred to as the peak alpha frequency (PAF), while the power associated with this specific frequency is termed the peak alpha power (PAP)^26^. PAF varies among individuals (around 10 Hz, equivalent to the period of 100 ms) and is correlated with different cognitive functions such as attention^27,28^, working memory^29^, and decision-making^30^. On the other hand, an interval of 100 ms forms a building block of immediate perception^31,32^. Visually identical stimuli presented in this interval are generally perceived as happening simultaneously^33^. Since this interval is equal to the period of an oscillator at the frequency of 10 Hz (i.e., circa PAF), it has been speculated that the internal clock’s pacemaker properties are related to this peak’s frequency: “Faster alpha rhythms would result in longer estimates of time than slower alpha rhythms, considering that more pulses would accumulate during the same physical time interval^34^.” The idea of the relationship between alpha oscillations to produce pacemaker pulses was proposed by Treisman^21–23^ and has been studied by researchers following Treisman’s work. For instance, Glicksohn et al. ^118^ (2009) considered the left–right asymmetry index for PAF, and Horr et al.^110^ discussed alpha band power to be related to time perception through attentional mechanisms. Samaha et al.^117^ also found a positive correlation between PAF and temporal resolution of visual perception.

To explore this relationship between alpha oscillations and time perception, besides investigating a plausible correlation between these two phenomena, we applied a noninvasive neuromodulation approach: transcranial alternating current stimulation (tACS). TACS is a transcranial electrical stimulation (tES) method, which passes a weak electric current, typically 1–2 mA, through the brain (via at least two electrodes, one of which is located on the studied area on the scalp). This current intensity can modulate brain activity regarding changes in the firing threshold in a population of neurons^35,36^. Studies have shown that tACS possibly drives the activity of cortical regions to the frequency of stimulation pulses, representing a significant entrainment of the brain’s cortical oscillations during and after the stimulation^37,38^. In the realm of transcranial brain stimulation techniques, such as TMS, tDCS, and tRNS, stimulating the occipital area on time perception has been explored (for a comprehensive review, refer to^6^). However, the utilization of tACS becomes pertinent when researchers aim to entrain brain oscillation frequencies toward specific target frequencies, typically serving as the independent variable. Therefore, tACS is recommended for inquiries seeking to assess the repercussions of modulating brain oscillations on cognitive functions^38^.The relationship between modulation of brain oscillations and time perception has been previously investigated in a handful of studies. Using a segregation/integration task, Ronconi et al.^111^ provided evidence for a causal relationship between the modulation of brain oscillations in the alpha band and the temporal resolution of perception through visual-auditory stimulation at frequencies around the alpha peak. They showed that the peak alpha frequency determines the integration/segregation of information such that higher frequencies lead to more segregation (two separated stimuli are perceived as two distinct stimuli). In comparison, lower frequencies cause the temporal integration (two separated stimuli are perceived as one continuous stimulus) of perceived stimuli. Thus, when the alpha peak is pushed to higher frequencies (but still within the alpha range, normally PAF−2 to PAF+2) through visual and auditory stimulation, the temporal resolution of perception (segregation) increases.

Regarding the use of tACS, Cecere et al.^112^ used a sound-induced double-flash illusion task to measure the duration of visual–auditory modality integration. In this task, a white disc appears on the gray background of a monitor for a very short time (12 ms), and a beep sounds simultaneously. A second beep sounded after a 36–204 ms delay. In the illusion, if the interval between the two beeps is small (less than what is known as the temporal window of illusion), the participant reports two flashes rather than one^39,40^. Appling tACS at three frequencies, PAF−2, PAF, and PAF+2, causally altered the time window of this cross-modal illusion (visual-auditory), such that stimulation at a higher (PAF+2)/lower (PAF−2) frequency decreased/increased the size of this window, increasing/decreasing the temporal resolution of perception. Battaglini et al.^105^ showed that the temporal binding window (TBW) with a non-alpha tACS stimulation (with 18 Hz) is no different from a sham-control condition. However, applying 10 Hz-tACS reduces the size of the temporal window of segregation. Venskus et al.^113^ used the double-flash illusion task to measure the TBW and the filled-duration illusion task with auditory modality to measure time perception. The authors’ initial assumption was that there would be a negative correlation between peak alpha frequency with time perception and the TBW. However, their results showed that although PAF had a positive relationship with TBW, it had no relationship with time perception. In addition, they found no relationship between alpha power and TBW. Mioni et al.^6,34^ used a time generalization task with a standard interval of 600 ms in the visual modality. They showed that applying tACS at these three frequencies (i.e., PAF−2, PAF, and PAF+2) affected the perception of duration. The authors concluded that the application of tACS did not cause changes in the dispersion of the results (better or worse temporal resolution) but led to a shift in the psychometric function, meaning that higher frequencies of tACS lead to shorter perceived intervals (and not less temporal resolution). Although these studies utilized different tasks, all focused on the occipital area with or without parieto-occipital and/or parietal areas for alpha peak extraction and tACS delivery to investigate temporal phenomena.

However, due to the far distance between the period of PAF (around 100 ms) and the one-second interval (1000 ms), studies showed less tendency to investigate this relationship; the studies tend to explore sub-second intervals, so the relationships between PAF/PAP and subjective one-second must be clarified. On the other hand, studies employ different tasks to tackle the problem, but a pure temporal task was rarely used. Here, we directly investigated the relationship between alpha peak characteristics and the pre-existing one-second perception.

## Materials and methods

### Participants

One hundred twelve students (50 women, 62 men, mean age = 24.30, SD = 4.49, 18–36 years old) volunteered to participate in this study through a public invitation at the University of Isfahan. We selected this age spectrum due to the consistency of the alpha peak in this period^44,45^. Experiment procedures were explained to the individuals over the phone. Those who agreed to participate in the study completed a pre-stimulus screening questionnaire for transcranial electrical stimulation^46^, which includes ten questions such as existing metal or electronic implants in the brain/skull or other parts of the body, surgical procedures involving the head or spinal cord, skin problems, epilepsy, and some other questions. Due to considering healthy participants, they also completed an Iranian version of the 28-item general health questionnaire (GHQ), including four criteria of somatic symptoms, anxiety/insomnia, social dysfunction, and severe depression^47^. Both questionnaires were filled out by telephone: Participants were instructed to set aside one hour to complete the questionnaires at their convenience, ensuring a solitary environment during the response period. Special attention was given to framing the questions in a manner that facilitated participants' thorough understanding. Those meeting the criteria for transcranial electrical stimulation were then directed to respond to the GHQ questions. Of those, 35 participants (mean age = 24.80, SD = 4.08, 18–36 years old) met the criteria required for tACS application and scored below 24 in the GHQ (screened as healthy, mean = 19.26, SD = 3.22, min = 12, max = 23). Twenty-four of these volunteers were randomly selected, but two were randomly replaced by two of the remaining 11 eligible participants because they did not have a clear peak in the EEG frequency spectrum. The final sample consisted of 24 participants (11 women, 13 men, mean age = 25.79, SD = 4.36, 18–36 years old). This number of participants was adopted due to 24(4!) counterbalanced order of stimulation, which also aligns with the previous studies (Supplementary Table 1).

All participants had normal or corrected-to-normal vision (with glasses) and signed a written and informed consent form to participate in the study. Each participation’s attendance in the Isfahan Cognitive Laboratory was adjusted according to their own schedule. All phases of the experiment were conducted between 8:00 a.m. and 2:00 p.m. The final sample had no dropouts (e.g., non-participation). The study was approved by the Research Ethics Committee of the University of Isfahan and performed in accordance with the Declaration of Helsinki.

### Procedure

**Figure 1:**
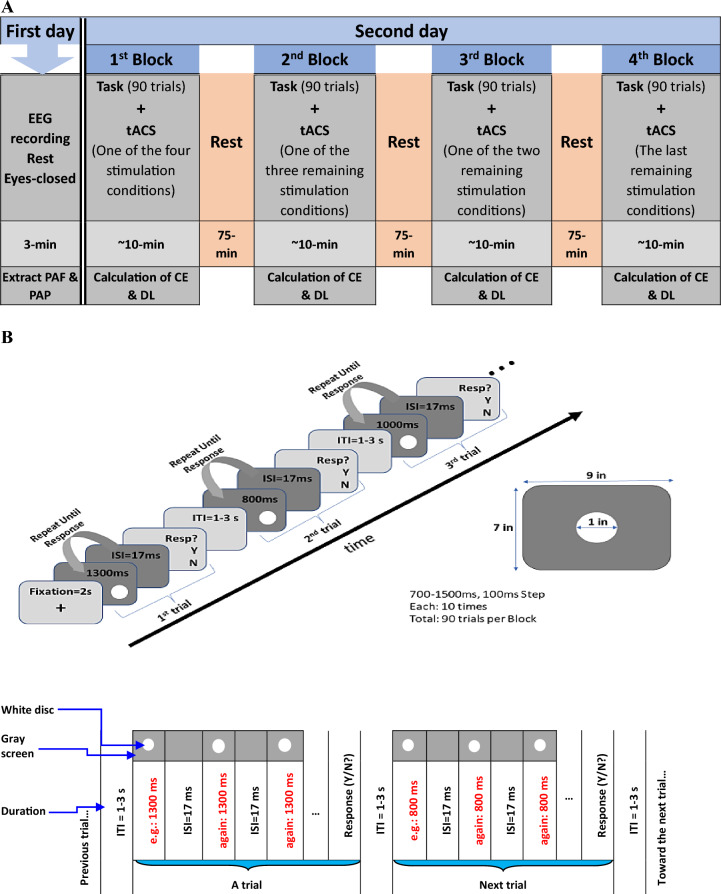
(**A**) Experiment timeline on the first and second days. TACS conditions comprise sham, PAF−2, PAF, and PAF+2. (**B**) Detailed illustration of the temporal task in two formats.

The participants attended the laboratory on two separate days. On the first day, a 3-min eyes-closed resting-state EEG was recorded for each participant. It took approximately 30 min to place the EEG cap on each participant’s head and an additional 10 min to remove it and wash their hair afterward. Following that, the PAF and PAP were extracted for each participant. It is essential to mention that there were no temporal tasks or tACS procedures conducted on the first day. On the second day, concurrent with tACS stimulation, each participant performed a time generalization task, comprising four blocks, each lasting around 10 min. The blocks included sham stimulation, as well as stimulation at PAF−2, PAF, and PAF+2 frequencies counterbalanced across 24 (4!) orders. These blocks were separated with a 75-min break (Fig. 1A).

Staying in the laboratory during breaks, participants were free to do what they wanted but were required to put aside their cellphones and watches to avoid looking at their second’s counter. They had access only to caffeine-free biscuits and juice, not tea or coffee, and were also not allowed to smoke cigarettes^48,49^. To prevent participants from practicing counting real clock seconds, none of them knew the details of the temporal task until the second day. The task was fully explained on the second day in the laboratory to ensure they understood it well.

### Temporal task

We selected a time generalization task in which the standard time interval is initially presented to the participant multiple times to encode it into memory. The task aims to measure human accuracy and precision in perceiving one second. Concerning the one-second interval, it is customary to divide brief intervals into sub- and supra-second categories^50^. Sub-second intervals involve more automatic processes, while supra-second intervals are more associated with high-level cognitive processes. In between, the one-second interval, which is at the heart of our study, is a culturally learned interval through clocks that remains arbitrary^51^. However, since the standard interval in this study is one second, already encoded into the memory in daily life, we did not present this pre-existing interval to participants. In the next step, comparison intervals are presented with a duration equal to or close to the one-second as the standard interval. Participants respond with yes/no, indicating whether the comparison interval is the same/different from the standard interval^52^. In the time generalization task, the responses (same/different) are non-directional in nature. Therefore, they resist response bias compared with tasks that rely on directional responses (i.e., longer/shorter)^53^.

The participants sat relaxed state in a quiet, dimly light room, positioned 60 cm from a screen (LED, vertical refresh rate = 60 Hz, Resolution = 1024 × 768, 9 × 7 inch^2^). The visual stimulus was a one-inch diameter white disc displayed at the center of the gray background. The “M” key, corresponding to the answer “same as one second”, was labeled “yes,” while the “N” key corresponding to the answer “different from one second” was labeled “no.” Participants were tasked with reporting whether the stimulus on the screen was present for exactly one second (same/different). Participants were explicitly told to report “no” if they recognized any difference, even if it was a slight deviation from one second. Participants were not shown the physical one-second interval before the start of the experiment; instead, they were asked to recall one second from their long-term memory based on their life experiences (i.e., pre-existing one-second). In this context, this task resembles the verbal estimation task, wherein participants must report the duration of the stimulation in conventional units (i.e., one second) but respond only by stating whether this is the same or different (without providing the numerical value of the stimulus duration in seconds). As this task relies on conventional temporal units, it is more pertinent than other methods (e.g., time reproduction) for probing the pacemaker component of the internal clock^54^.

A significant challenge arises from the random fluctuations in discrimination performance from one moment to another. For instance, there are occasions when a specific physical difference between two stimuli is perceived, while on other occasions, this difference is not perceived^55^. Therefore, to enhance the results, the single stimulus (e.g., 800 ms) was presented multiple times (with the same duration, i.e., 800 ms) until the participant decided on a response (in this case, “no”)^56,57^. In other words, in a trial, the participants were not exposed to the white disc just one times but repeatedly (i.e., they were shown the disc twinkling with a constant period of circa 1000 ms) (Fig. 1B). Participants were informed that in a given trial, the white disc would repeatedly turn on and off at a stable rate and that this rate remains constant during all repetitions in the trial until response but may change in the subsequent trial. Participants had the liberty to respond after any number of stimulus repetitions as they wanted, but they were encouraged to provide answers promptly (e.g., after 3 or 4 representations). These measures were implemented to minimize the potential impact of distractions and fatigue on the experiment. The inter-stimulus interval (ISI) was adopted to one screen frame (ISI = 1/60 s ~ 17 ms), as verified by an oscilloscope (Supplementary Fig. 1), and was perceptible to participants^58^. This brief yet discernible ISI was chosen to mimic the temporal dynamics of the second hand of a real clock. After pressing the response key (yes/no), the twinkling stimulus disappeared for a random period between 1 and 3 s (equivalent to the inter-trial interval; ITI) before initiating the next trial. The stimulus was then presented again with another comparison interval (e.g., 1300 ms) and started twinkling until a response was received. This cyclic process continued similarly for other comparison intervals.

Comparison intervals were initially set at 500, 600, 700, 800, 900, 1000, 1100, 1200, 1300, 1400, and 1500 ms. These intervals were selected to create symmetry around the 1000 ms (i.e., standard interval). Interval lengths are typically chosen in proximity to the standard interval to ensure that the number of “yes” responses is close to zero at both ends of the temporal spectrum^55^. A pilot study involving 9 participants (five men, distinct from 24 participants of the study) revealed infrequent selections of 500 and 600 ms (Supplementary Table 2). Consequently, these two intervals were excluded from the task to reduce the experiment time and fatigue, which could adversely impact the results. Trials were presented randomly, with each interval appearing in ten trials per block. Thus, each block comprised 90 trials (nine intervals, each presented ten times). Participants were unaware of the exact number of one-second intervals within a block (i.e., 10/90 ~ 11% of all trials). All phases of the temporal task were designed using PsychoPy v2022.2.5 software^59^.

Regarding temporal perception, the two critical dependent variables are accuracy and precision of time perception^60^. This means that the closer the perceived time (subjective) is to the physical time (objective), the higher the accuracy of the results, and the lower the variability of perceived time, the higher the precision and temporal resolution. In psychophysics, the parameters suitable for studying time perception are constant error (CE) and difference limen (DL)^3,55,61^. CE is the difference between perceived time (i.e., point of subjective equality; PSE) and actual time (i.e., point of objective equality; POE) (i.e., CE = PSE−POE). The closer the time perceived by the subject (i.e., PSE) to the real-time of the phenomenon (i.e., POE), the higher the accuracy of the perceived duration. On the other hand, DL (= 0.6745SD, where SD stands for the standard deviation) serves as a measure of the threshold or sensitivity to discriminate between two temporal intervals. A smaller DL value indicates less scattered results, suggesting a higher precision in time perception. Put differently, a reduced DL signifies an enhanced ability to detect even subtle differences in time intervals, contributing to a finer resolution in temporal discrimination. Therefore, in the context of studying time perception, a lower DL is indicative of heightened precision and a more refined capacity to discern temporal distinctions^55^.

The temporal task was developed in PsychoPy in such a way that the number of “yes” responses corresponding to each interval was stored in a separate text file. Using these responses in the waveform moment analysis (WMA)^62^ in MATLAB R2022b (www.mathworks.com), CE and DL were extracted for each block of each participant^55^.

### EEG recording and analysis

**Figure 2:**
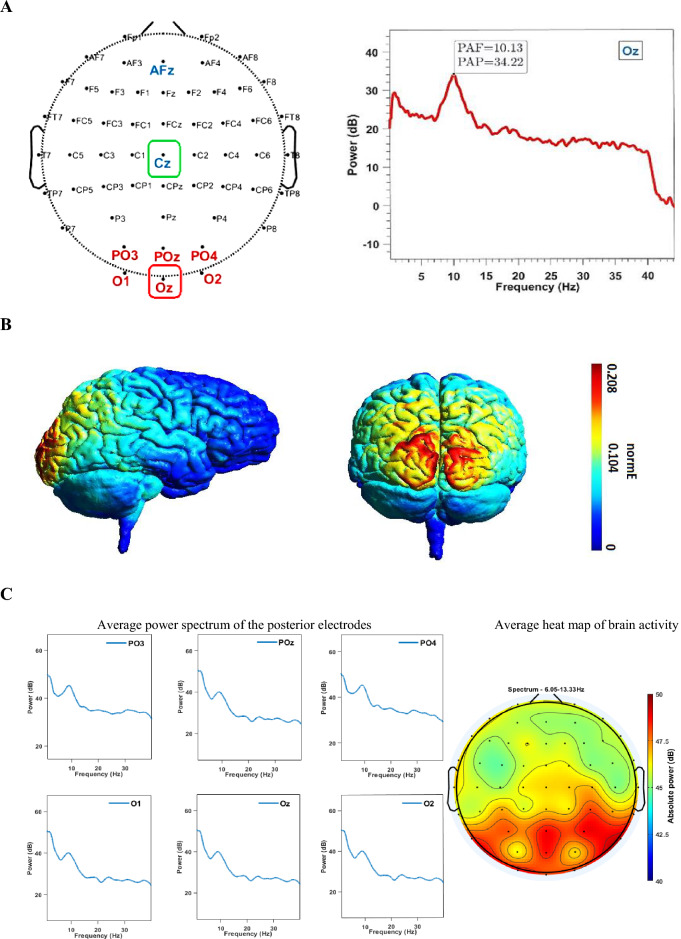
(**A**) (Left) Configuration of the 54 scalp electrodes for EEG recording based on the 10–10 international system; AFz as the ground electrode, Cz as the online reference electrode, with electrodes utilized for alpha peak extraction highlighted in red—during tACS; Cz as reference electrode and Oz as target electrode. (Right) An example of PAF and PAP extracted from a participant's Oz electrode, where PAF represents the horizontal and PAP represents the vertical component of the frequency distribution of EEG in Fourier space. (**B**) Simulation depicting the normalized electric field delivered by tACS following our protocol, simulated using SimNIBS, highlighting the maximal induced field over the posterior cortex. (**C**) (Left) The average power spectrum of all participants in the posterior area indicates the alpha peak. The horizontal and vertical axes represent frequency (Hz) and power (dB), respectively. (Right) The average heat map of brain activity from PAF_min_−2 (6.05 Hz for participant number 9) to PAF_max_+2 (13.33 Hz for participant number 16) for all participants (eyes-closed resting state) reveals heightened activity in the posterior area.

EEG data were recorded from the participants using a 64-channel EEG system with passive electrodes (EEG5000Q, CMRR > 110 dB, 54 scalp channels, Negar Andishgan Co. Ltd.) at a sampling rate of 500 Hz. The electrodes were placed according to the international standard 10–20 system (Fig. 2A, Left). Each participant underwent a 3-min eyes-closed resting-state EEG recording while the impedance was kept below 20 kΩ. The online filter was 0.5–70 Hz, and AFz and Cz electrodes were adopted as the ground and reference electrodes, respectively. For offline pre-processing using EEGLAB version v2021.0^63^, a high-pass filter of one Hz and a low-pass filter of 40 Hz were applied. Electrodes were then re-referenced to the common average^64^, and the ASR (spherical splines) method was employed to check noisy parts and channels ass recommended by EEGLAB. Subsequently, some signal parts were removed manually, and noisy channels were interpolated (mean = 1.92, SD = 2.89). Then, with the help of ICA (Runica algorithm), components lacking a 1/f pattern (mean = 3.88, SD = 4.41) were manually removed^65^, followed by baseline correction was performed (Supplementary Fig. 2). Following this, the power spectra of the posterior electrodes were plotted employing EEGLAB channel time–frequency defaults [sub-epoch: the length of the signal, wavelet cycle: (min = 3, max/fact = 0.8), 200 time points, limits: Padding1, divisive baseline (DIV)] for better frequency resolution (Δf = 0.06 Hz) (Fig. 2A, Right). Finally, due to the non-normal distribution of PAF and PAP across the six desired posterior electrodes (O1, O2, Oz, PO3, PO4, and POz), we opted for the weighted median (instead of the weighted average) in the calculation of PAF and the median (instead of the average) for calculating PAP.

### tACS

NeuroStim2 (Medina Teb) was utilized for tACS delivery. Sponge-covered rubber electrodes moistened with saline were centrally placed on the Cz (11.5 cm × 8.5 cm ~ 98 cm^2^) as the reference and the Oz (4.5 cm × 4.5 cm ~ 20 cm^2^) as the target point, according to the international standard 10–20 system and fixed by elastic bands (Fig. 2A, Left). Due to the large size of electrodes, tACS is a non-focal device which result in contaminating expanded area (i.e., parietal and posterior area) with the electric current. As the posterior area was the desired target, the smaller electrode was placed on this area. The peak-to-peak current intensity was chosen to be 1.6 mA. Thus, the posterior area was targeted with a current density of 79 μA/cm^2^, and the reference area was targeted at a current density of 16 μA/cm^2^. Given that the minimum required current density for effective stimulation is 17 μA/cm^2^, the posterior area was stimulated with a current density above the threshold. In contrast, the reference region was targeted for stimulation with a current density below the required threshold^66^. It is worth noting that this is the current direction, not the current density, that alternates between the two electrodes. 140 mM saline was consistently applied to the sponges to avoid skin irritation caused by electrical stimulation^67^. However, a small amount of saline was added during the task to avoid potential interruption of electric current^68^. The stimulation duration in each block equaled to the duration of the 90 trials of the temporal task, approximately ten minutes, including a 20-s ramp-up and a 20-s ramp-down in both sham and real stimulations.

Each participant engaged the temporal task across four random blocks, comprising three stimulation conditions at PAF−2, PAF, and PAF+2 frequencies and the sham condition, resulting in a total real stimulation duration of 30 min. Adopting PAF−2 and PAF+2 was in accordance with the literature which usually define these frequencies as the boundaries (off-peaks) of the alpha band. By using PAF as an anchor point, the tACS frequencies for each participant were adopted based on their individual PAF.^69–72^. As the effects of tACS stimulation in the alpha band may last up to 70 min, blocks were separated by a 75-min break (Fig. 1A)^73^. Participants were asked to report phosphenes^74^ at the beginning of each stimulation, but none reported observing any. After each block, participants were asked to report any side effects in the post-stimulation questionnaire^75^. The SimNIBS-3.2 toolbox was applied to investigate the efficacy of the electric field produced by tACS through the posterior area under our protocol (I = 1.6 mA, Oz electrode = 20 cm^2^, Cz electrode = 98 cm^2^)^76^.

### Statistical analyses

The values of CE and DL were extracted for each participant across four blocks including real PAF−2, PAF, PAF+2 stimulations, and sham condition, resulting in a total of 96 CE and 96 DL measurements (i.e., 4 blocks × 24 participants). The waveform moment analyses (WMA) were used for this purpose. In this method the $f_{i}$ is considered to be the reported relative frequency of ‘yes’ responses associated with comparison interval $c_{i}$(i.e., number of ‘yes’ responses to $c_{i}$ divided by ten related trials of a block). Initially, these frequencies are transferred into a probability distribution by the transformation $p_{i} = \frac{{f_{i} }}{{\sum f_{i} }}$, where $i$ = 700–1500 ms with 100 ms steps. Then, considering the standard interval of one second, the CE and the DL are calculated as $CE = - 1 + \Sigma p_{i} c_{i}$ and $DL = 0.6745\sqrt {\sum p_{i} \left( {c_{i} - CE - 1} \right)^{2} }$. Ready MATLAB scripts are available at^77^ in^55^. This technique has been applied in other studies investigating temporal discrimination^78,79^.

The data were processed and statistically analyzed hereafter using IBM SPSS Statistics (version 26). GraphPad Prism (GraphPad Software, Version 9.5.1 (733) for Windows, San Diego, California, USA, www.graphpad.com) and Microsoft Excel (Microsoft Corporation (2018). Microsoft Excel. Retrieved from https://office.microsoft.com/excel) were utilized to generate graphs for better visualization. In the initial step, we assessed outliers and the normality of data, including the Shapiro–Wilk test and Q-Q plot visual inspection. Then, the collected data were divided into two parts for the primary analyses. The first part focused on examining the correlation between peak alpha frequency (PAF) and peak alpha power (PAP), extracted on the first day, with CE and DL extracted on the second day. For this purpose, separate Pearson correlation analyses were performed, and a false discovery rate (FDR) analysis using the Benjamini–Hochberg procedure was applied to control the multiple comparison error. These analyses aimed to understand the plausible correlation between the alpha oscillations and time perception.

Applying repeated-measures ANOVAs, the second part of the analysis aimed to compare the effects of tACS in four different stimulation conditions. These conditions comprised the sham condition, where no stimulation was applied, as along with three real tACS conditions: PAF−2, PAF, and PAF+2. These were considered as within-subjects factors, assessing their impact on time perception.

## Results

The average power spectra across all participants, captured under the six desired electrodes, are depicted in Fig. 2C (Left). Additionally, Fig. 2C (Right) portrays the mean heat map of brain activity from PAF_min_−2 (6.05 Hz) to PAF_max_+2 (13.33 Hz) for all participants, revealing higher activity in the posterior region. Given the applied age range (i.e., 18–36 years), age did not serve as a predictor of either PAF (r = 0.320, p = 0.127) and PAP (r = 0.247, p = 0.245), aligning with expectations^80^ (Supplementary Fig. 3, Top). Concerning tACS, the SimNIBS-3.2 toolbox^76^ confirmed that 1.6 mA electric current is a sufficient flow passing through the posterior area while the centers of two electrodes were located at Oz (20 cm^2^) and Cz (98 cm^2^) (Fig. 2B). The post-stimulation questionnaire^75^ showed that participants were unable to distinguish the sham stimulation from real stimulations above chance (correct answers < 25%). Besides, participants tend to respond to comparison intervals closer to the standard interval (i.e., one second) after more repetition (4.15 times for 1000 ms) and time (4.22 s for 1000 ms) (Supplementary Fig. 3, Bottom). This result was predictable because decreasing the difference between stimuli increases response time^81^.

**Figure 3:**
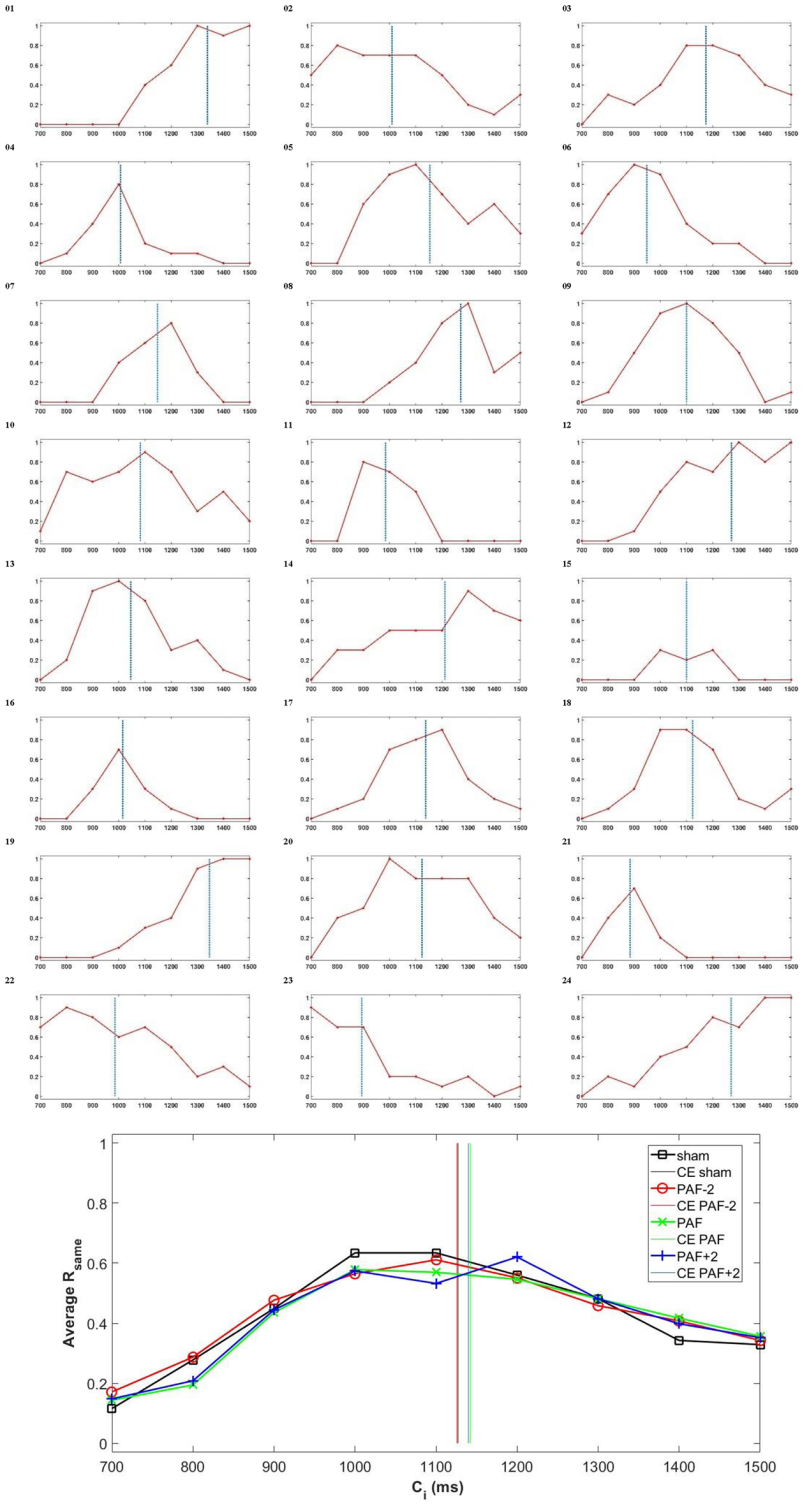
(Top) Relative frequency of 'yes' responses (i.e., judging the comparison interval as one second) for the sham condition among all participants. The dotted vertical line denotes the PSE estimate derived from waveform moment analysis. (Bottom) The average relative frequency of ‘yes’ responses for all stimulation conditions, including tACS at PAF−2, PAF, PAF+2, and sham. Vertical lines indicate PSEs.

After employing WMA, each participant’s CE and DL for four tACS conditions were calculated (Fig. 3), and an outlier analysis was performed, but no outliers were identified. Due to our relatively small sample size, we assessed the distribution of variables to determine an appropriate statistical method. The Shapiro–Wilk test indicated no evidence of non-normality (W > 0.94, p-value > 0.19 for all ten variables, including PAF, PAP, and CE, DL in the sham, PAF-2, PAF, and PAF + 2 tACS conditions). Additionally, Q-Q plots were visually inspected to validate the normality assumption (Supplementary Fig. 4). Based on these results and visual assessments, we deemed a parametric test to be appropriate for further analyses. The data of PAF, PAP, CE, and DL for all four tACS conditions is gathered in Table 1A.

**Table 1:**
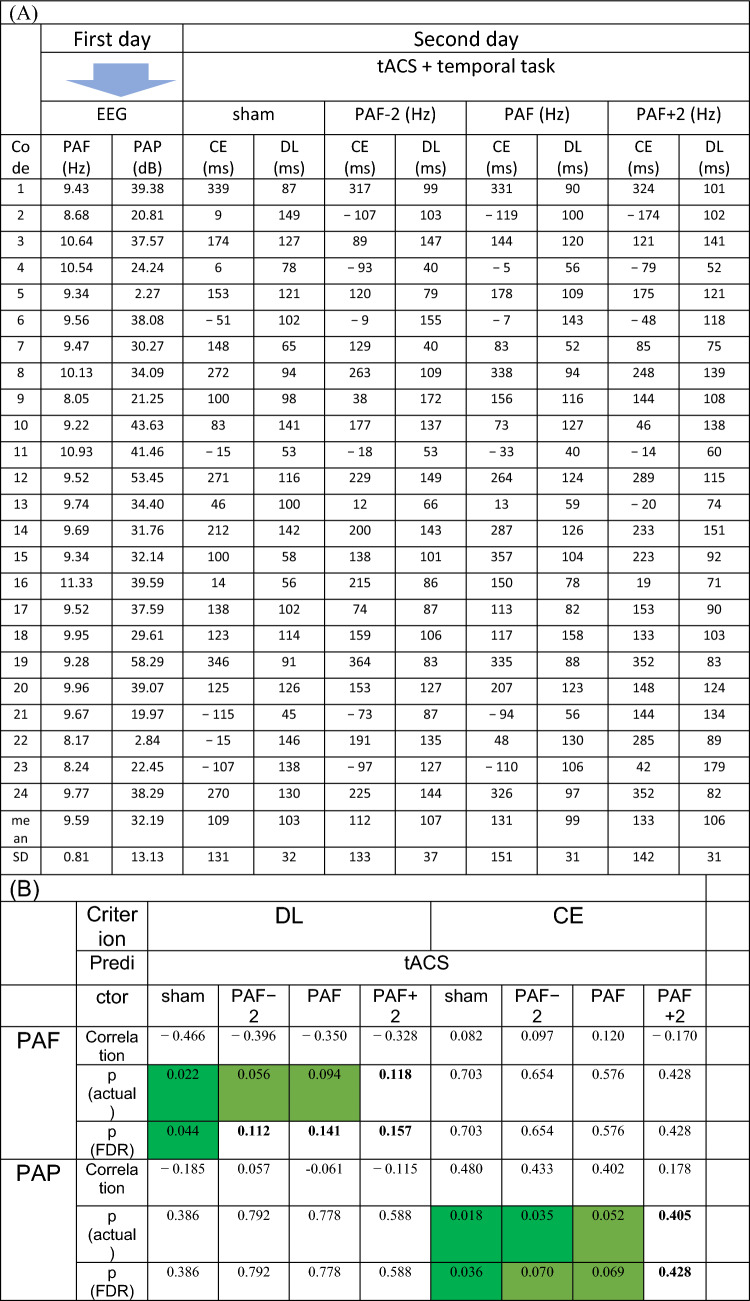
(A) Participant data featuring alpha peak and time perception parameters across four stimulation conditions.

(B) Pearson correlations with corresponding significances for different stimulation conditions. Significant results (p < 0.05) are highlighted in dark green, while marginally significant results (0.05 < p < 0.10) are shaded in pale green. It is noteworthy that as the stimulation frequency increases, the significance levels tend to decrease.

We conducted Pearson’s correlation tests to examine potential associations between PAF and PAP with CE and DL.

**Figure 4:**
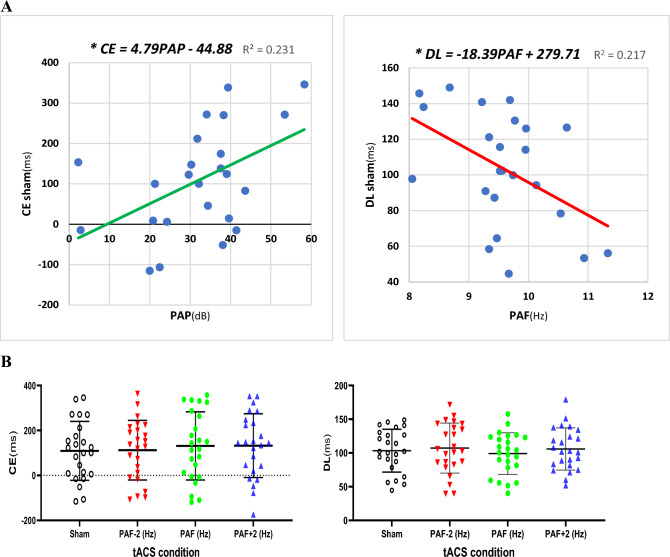
(**A**) The best-fitted line for the correlation between PAP and CE (left) shows a positive slope, indicating that participants with higher PAP tend to report longer intervals as one second. Conversely, the best-fitted line for the correlation between PAF and DL (right) has a negative slope, indicating that participants with higher PAF are more precise in distinguishing their one-second interval (i.e., PSE) from adjacent intervals. (**B**) Repeated measures ANOVAs revealed no effect of tACS on CE (left) (F(3, 69) = 0.950, p = 0.421, partial η^2^ = 0.040) and DL (right) (F(3, 69) = 0.741, p = 0.532, partial η^2^ = 0.031). * p < 0.05.

Considering the “sham” condition, the correlation analysis revealed a non-significant relationship between CE and PAF (r = 0.082, p = 0.703) and between DL and PAP (r = − 0.185, p = 0.386). In contrast, the correlation analysis between CE and PAP revealed a significant positive relationship (r = 0.480, p = 0.018, FDR-corrected p = 0.036). CE (ms) increased moderately with higher PAP (dB) values, as indicated by the linear relationship in CE = 4.79PAP–44.88 (R^2^ = 0.231). Specifically, CE reached its root at PAP = 9.37 dB and increased by 4.79 ms for each dB increase in PAP (Fig. 4A, Left). Additionally, the correlation analysis between DL and PAF revealed a significant negative relationship (r = − 0.466, p = 0.022, FDR-corrected p = 0.044). The linear relationship between PAF (Hz) and DL (ms) was obtained as DL = − 18.39PAF + 279.71 (R^2^ = 0.217). The negative correlation between DL and PAF indicates that DL tends to decrease with higher PAF values: DL decreases by 18.39 ms for each Hz increase in PAF (Fig. 4A, Right). Finally, Pearson correlation tests were also conducted to investigate the correlation between PAF and PAP with CE and DL across the three “real” stimulation conditions. These analyses revealed non- or marginally significant associations, particularly after FDR-correction, as summarized in Table 1B. In addition, the correlation between each electrode’s PAF and PAP with each CE and DL is presented in the Supplementary Table. 3.

In the final step, we conducted repeated-measures ANOVAs to assess the impact of different stimulation conditions, including PAF−2, PAF, PAF+2, and sham, on CE and DL. The results revealed no significant changes in CE (F(3, 69) = 0.950, p = 0.421, partial η^2^ = 0.040) and DL (F(3, 69) = 0.741, p = 0.532, partial η^2^ = 0.031) across the four conditions. These findings in repeated-measures ANOVAs suggest that the different tACS conditions applied in this study did not lead to significant alterations in participants’ CE and DL during the time generalization task (Fig. 4B).

To ensure our sample size adequacy, post hoc power analyses using G*Power (version 3.1.9.4^82^) indicated 22 (CE-PAP) and 22 (DL-PAF) total sample size for correlation relationships and 4 (CE) and 6 (DL) total sample size for ANOVAs. These results affirm the study’s statistical power to detect observed effects and it means our sample size (N = 24) was an appropriate number for this study.

## Discussion

Building on existing evidence, we aimed to shed new light on the neural underpinnings of time perception. The primary objective of our study was to directly investigate the existence of a correlation between the parameters of peak alpha and the accuracy and precision of one-second perception. Furthermore, we sought to explore a potential causal relationship by examining whether modulating this peak through tACS could influence the accuracy and precision of perceived time. These inquiries were designed to provide valuable insights into the neural oscillations that may play a role in human temporal discrimination ability.

In the absence of brain stimulation (i.e., sham condition), our study revealed no correlation between DL and PAP, but a negative correlation was observed between DL and PAF during the one-second generalization task. These results suggested that higher peak frequencies of neural oscillations in the alpha band corresponded to lower variability in time perception, leading to greater precision and resolution in perceiving one second. In contrast, CE did not exhibit any significant correlation with PAF but positively correlated with PAP. When PAP values were greater/less than 7.67 dB, individuals reported the subjective second as greater/less than the objective second. These findings suggest that PAF and PAP play a crucial role in shaping the subjective perception of one-second time intervals.

In comparison to previous studies, we obtained similar, different, and new results. Glicksohn et al.^118^ (2009) observed a significant positive correlation between the left–right asymmetry index for PAF (but not the values of PAF and PAP) and time perception. Additionally, their study employed a time production task, which might have been susceptible to higher errors^33^ with 4, 8, 16, and 32 s intervals. However, we employed a time generalization task with one second as the standard interval. Finally, their PAF data were extracted from P3 and P4 regions rather than the posterior electrodes used in our study. Samaha et al. (2015) employed a two-flash fusion task to measure the temporal resolution of visual perception. The blank intervals were 10–50 ms between a pair of 40 ms-visual stimuli. They found that individuals with higher alpha frequencies have vision with finer temporal resolution concurrent with our study. Cecere et al.^112^ and Ronconi et al.^111^ investigated time perception with different tasks and time intervals, finding that higher frequencies correspond to higher temporal resolution. Cecere et al.^112^ utilized a sound-induced double-flash illusion task, while Ronconi et al.^111^ employed a segregation/integration task for time intervals close to the alpha-hand period (approximately 100 ms). Venskus et al.^113^ distinguished between the temporal binding window and time perception, interpreting PAF as related to the temporal binding window but not time perception. In contrast, Battaglini et al.^105^ delivered tACS at 10 Hz (but not PAF) to the V5/MT area of the right hemisphere (but not V1), resulting in a decline in temporal resolution compared to sham and 18 Hz tACS. However, they did not modulate PAF or other frequencies within the alpha band. Mioni et al.^6,34^ employed a time generalization task with a standard interval of 600 ms, and they found that entrainment of the alpha peak did not affect temporal resolution (related to DL) but did shift the psychometric function (related to CE). An important consideration is that using conventional intervals, such as the second in our task, can provide advantages as these intervals are likely encoded in the brain due to long-term memory (i.e., pre-existing interval). Nonconventional intervals like 600 ms may not be as stably encoded or as easily compared to conventional intervals, therefore the examiner should present the nonconventional standard interval to the participants before presenting comparison intervals. Consider that our study showed that PAF correlated with DL, while PAP correlated with CE. This differs from Mioni et al.’s findings, where PAF modulation affected CE but not temporal resolution. All in all, we urge that alpha oscillations’ crucial role in sub-second time perception (abovementioned studies) can be generalized to one-second time perception despite this interval being far from the period of alpha frequency.

In terms of models, our results can be explained by internal clock model mechanisms as the alpha peak characteristics can be considered as the vital parameters of the clock stage of SET by playing role in estimation of pre-existing one second. Frequency (PAF) and power (PAP) of alpha peak may determine the underpinning mechanisms of clock stage in time perception as these properties determine the precision and accuracy of one-second time perception, respectively. Alternatively, these results can be described by intrinsic time perception models, which suggest the overall activity of the brain (probably in a specific phase of synchronization, here alpha band) can present an internal sense of time^8^. Particularly, alterations in alpha waves may impact a broad range of brain regions responsible for constructing functional brain networks, such as the visual network, frontoparietal network, and default mode network^83–86^. These network modifications can alter state-dependent networks potentially involved in time perception^16,87^, or influence neural information processing, thereby changing our internal sense of time passage^15,88^.

Despite the intriguing correlation findings, our attempts to establish causal relationships by modulating the alpha peak using tACS were unsuccessful. The lack of effect of tACS on time perception in this experiment may have been due to using a conventional tACS device, which is a non-focal device. In this case, an HD-tES device is recommended to achieve the effects of alpha peak modulation on time perception because these devices generate a stronger electric field in the cortex with higher focal power^89,90^. It is also possible to use the same laboratory settings, but other tools (e.g., rhythmic sensory stimulation around PAF) can be used to modulate alpha peak frequency and its effect on one-second time perception^35^. Further investigation into the factors influencing tACS modulation and potential limitations encountered during the tACS experiments would be valuable for future studies.

Although the administration of tACS did not yield a discernible impact on the observed outcomes (i.e., CE and DL, using ANOVAs, as indicated in the preceding paragraph), it undermined the correlations’ significance between CE and PAP, as well as between DL and PAF, as illustrated in Table 1B. More precisely, the outcomes reveal a regular increase in p-values and a regular decrease in Pearson correlation coefficients through the sham condition to PAF−2, PAF, and PAP+2, corresponding to an escalation in stimulation frequency. We conjecture that this phenomenon may be attributed to the activation of additional brain regions prompted by the stimulation of the posterior area. Several investigations employing diverse methods such as behavioral assessments, neuroimaging, and electrophysiology have suggested the involvement of various brain regions in temporal processing. These encompass the basal ganglia, cerebellum, supplementary motor area, premotor area, parietal cortex, occipital cortex, and dorsolateral prefrontal cortex^91–98^. The dynamic contribution of these interconnected structures to temporal information processing is not uniform; it is contingent not only upon the duration of the time interval under consideration but also on the cognitive framework implicated in the specific task and the sensory modality utilized for time delineation^6^. Given the interconnected nature of the brain and its operation as a network of regions, stimulating one area may instigate activity in other areas^99^. These additional regions may, in turn, contribute to the complex network dynamics involved in time perception and/or other pertinent cognitive functions such as attention. Consequently, we propose exploring the stimulation of other brain areas to investigate their potential roles in shaping one-second time perception, as elucidated in this study. Finally, functional connectivity and brain network analysis serve as essential tools for future studies. They offer a comprehensive approach to unraveling the complexities of the brain’s functional organization and provide valuable information on the neuronal underpinning of time perception^98,100^.

We employed resting state eyes-closed EEG since the alpha oscillation is commonly more pronounced in this condition compared to the eyes-open condition and, notably, during task engagement^101^. However, as a suggestion for future studies, one can assess the possible relationships between other EEG characteristics (such as power and functional connectivity) instead of the alpha peak, which can be assessed in both rest and task performance EEG recording. Furthermore, one can assess alpha band (and also other EEG) characteristics during the critical interval (i.e., from stimulus presentation to the participant’s response) by epoching the EEG signals based on this period. This targeted analysis may reveal significant correlations between ongoing alpha (and also other brain) oscillations and time perception features.

Attention plays a pivotal role in time perception^41^. In the attentional-gate model (AGM)^42,43^, attention directed towards temporal aspects influences judgments regarding prospective duration. The AGM introduces an attentional gate positioned between the pacemaker and the counter, controlling the flow of pulses. More pulses are accumulated when heightened attention is directed towards the passage of time, particularly in the absence of attentional resources allocated to a concurrent non-temporal secondary task (as in the case of a dual-task paradigm)^33^. Given our exclusive focus on time in this study, without any concurrent task, participants dedicated all their attentional resources to temporal considerations. In terms of memory, increasing working memory (WM) load consistently reduced subjective duration, with this effect scaling with durations^102^. However, our task imposed minimal memory demands. Participants were simply asked to remember the duration of a regularly perceived standard one-second interval^103^. In contrast, many tasks required participants to encode various intervals and subsequently retrieve them from memory (higher memory demands). Thus, our tasks involved lower memory demands. Regarding brain oscillations, given that delta-to-theta (∼ 2–8 Hz) oscillations over frontal brain regions coordinate alpha oscillations over the visual cortex during both the initiation and transition of representational states in visual working memory when executing multitask sequences^104^, we suggest that researchers explore the impact of this phenomenon on the perception of pre-existing visual one-second interval. Indeed, disentangling the roles of attention and working memory from time perception remains a significant challenge^102^.

Some critics may argue that using a periodic stimulus in our research could lead to the entrainment of brain oscillations. However, we carefully considered the frequency of the periodic visual stimulus used in our study, which was approximately one hertz (ranging from 0.67 Hz for 1500 ms to 1.43 Hz for 700 ms). In contrast, brain oscillations in the alpha region typically range from 8 to 12 Hz. The visual stimulus frequency (around one hertz) was far from the alpha region (around 10 Hz) to minimize the likelihood of resonance and entrainment of brain oscillations in the alpha range. Similar studies have shown that sensory rhythms far from the alpha region are unlikely to affect the alpha rhythm and time perception^105^. In fact, just some specific frequencies far from one hertz can affect brain oscillations^106^. For a more comprehensive understanding of this topic, readers can refer to the work by Norcia et al. (2015), which provides valuable insights into the relationship between sensory stimulation and brain oscillations. In conclusion, the distinct distance between the visual stimulus frequency and the alpha range used in our study provides confidence in the reliability of our results concerning time perception and alpha oscillations.

In the realm of time perception, heartbeats present an intriguing aspect to consider. While Schwarz et al.^116^ argue that heart rate may not significantly impact time perception, Pollatos et al.^115^ propose that this physiological parameter plays a role in shaping our perception of time. Recent studies have further shed new light on this connection, demonstrating that a slower pre-stimulus heart rate^107^ and stimulus presentation during the relaxation period of the heart^108^ result in longer duration estimates in the milliseconds range. Given that the frequency of heartbeats approximates one hertz^109^, which is remarkably close to the time intervals examined in our study, it is plausible to consider heart rate as a potentially influential factor due to its resonance with our perception of one-second intervals. Furthermore, the link between heart rate and arousal suggests an impact on the speed of the internal clock’s pacemaker^3^. Interestingly, peak alpha frequency and heart rate might share a common foundation, as discussed comprehensively in Klimesch^114^. Consequently, we recommend recording participants’ heartbeats as another relevant parameter in future studies focused on one-second tasks.

Lastly, we highly recommend extending this temporal task to include other sensory modalities, particularly the auditory domain. By doing so, we can investigate whether the observed correlation between alpha peak parameters and time perception holds across different modalities. This exploration will help ascertain whether the alpha peak and time perception relationship are independent of the sensory modality involved. Understanding the generalizability of these findings across modalities can provide valuable insights into the fundamental mechanisms governing time perception and its neural underpinnings. Additionally, such an approach can contribute to a more comprehensive understanding of how the brain processes temporal information, allowing for a more holistic perspective on the intricate interplay between alpha oscillations and time perception. Future studies incorporating multiple sensory modalities will undoubtedly enrich our knowledge in this field, bringing us closer to a more unified understanding of temporal cognition.

## Conclusion

Regarding one second, the precision and accuracy of time perception are correlated with peak alpha frequency and peak alpha power, respectively. Our attempt to modulate the alpha peak by tACS to manipulate time perception failed. Employing HD-tACS and stimulating other regions of the cortex are recommended to overcome the obstacle, resulting in a significant effect of tACS.

### Supplementary Information

Supplementary Information.

## Data Availability

Correspondence and requests for materials should be addressed to Mahgol Tavakoli.
