# Supplementary material for: Exploring the correlation and causation between alpha oscillations and one-second time perception through EEG and tACS
Source: Sci Rep. 2024 Apr 5;14:8035. doi: 10.1038/s41598-024-57715-6 (PMC10997657; doi:10.1038/s41598-024-57715-6)
Supplement: Supplementary file 1 — Supplementary Information. [file 41598_2024_57715_MOESM1_ESM.pdf]

Supplementary materials

Exploring the Correlation and Causation Between Alpha Oscillations and One-Second Time Perception Through EEG and tACS

Ehsan Mokhtarinejad
Department of Psychology, Faculty of Education and Psychology, University of Isfahan, Isfahan, Iran
e.mokhtarinejad@edu.ui.ac.ir

Dr. Mahgol Tavakoli
Department of Psychology, Faculty of Education and Psychology, University of Isfahan, Isfahan, Iran

m.tavakoli@edu.ui.ac.ir

Dr. Amir Hossein Ghaderi
Department of Psychology, Faculty of Education and Psychology, University of Isfahan, Isfahan, Iran

Center for Affective Neuroscience, Development, Learning and Education, University of Southern California (USC), Los Angeles, USA
ghaderia@usc.edu

Table 1. Participant demographics: The counterbalanced stimulation order is presented in the last column.

Code	Gender	Age	GHQ Score	Stimulation Order
01	Female	22	19	sham, PAF-2, PAF, PAF+2
02	Female	21	20	sham, PAF-2, PAF+2, PAF
03	Male	28	20	sham, PAF, PAF-2, PAF+2
04	Male	36	22	sham, PAF, PAF+2, PAF-2
05	Male	22	22	sham, PAF+2, PAF-2, PAF
06	Male	23	23	sham, PAF+2, PAF, PAF-2
07	Female	30	19	PAF-2, sham, PAF, PAF+2
08	Male	26	12	PAF-2, sham, PAF+2, PAF
09	Female	23	16	PAF-2, PAF, sham, PAF+2
10	Male	29	23	PAF-2, PAF, PAF+2, sham
11	Female	28	22	PAF-2, PAF+2, sham, PAF
12	Female	30	20	PAF-2, PAF+2, PAF, sham
13	Male	24	19	PAF, sham, PAF-2, PAF+2
14	Male	25	15	PAF, sham, PAF+2, PAF-2
15	Male	27	23	PAF, PAF-2, sham, PAF+2
16	Female	21	21	PAF, PAF-2, PAF+2, sham
17	Male	35	21	PAF, PAF+2, sham, PAF-2
18	Female	27	23	PAF, PAF+2, PAF-2, sham
19	Female	21	16	PAF+2, sham, PAF-2, PAF
20	Male	23	21	PAF+2, sham, PAF, PAF-2
21	Male	26	19	PAF+2, PAF-2, sham, PAF
22	Female	18	20	PAF+2, PAF-2, PAF, sham
23	Female	26	18	PAF+2, PAF, sham, PAF-2
24	Male	28	16	PAF+2, PAF, PAF-2, sham

Table 2. Percentage of the participants' 'yes' answers to the temporal task in the pilot study.

Comparison interval	500ms	600ms	700ms	800ms	900ms	1000ms	1100ms	1200ms	1300ms	1400ms	1500ms
Proportion of "yes" answers	0.00%	1.67%	10.00%	18.33%	33.33%	51.67%	78.33%	55.00%	48.33%	21.67%	10.00%

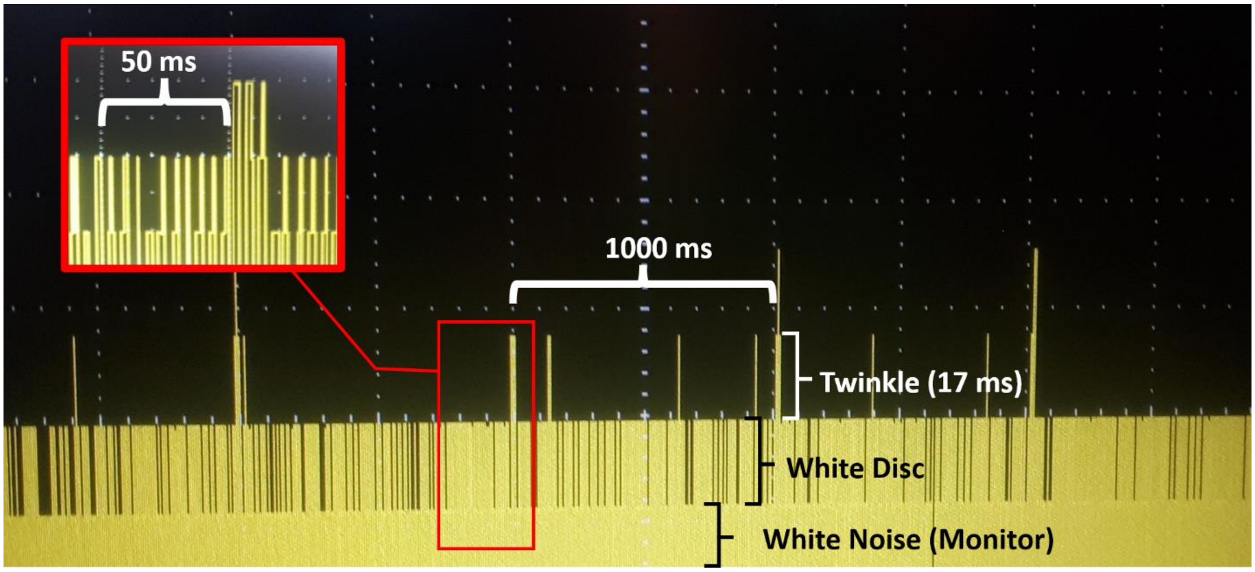

Figure 1. The connection of a photodetector (Leader Laser Power Meter, Model LPM-8000) to an oscilloscope (GW Instek GDS-1054B) was established with the sensor positioned on the white disc (the stimulus). The recorded data unveiled that the white disc's twinkle duration is approximately 17 ms.

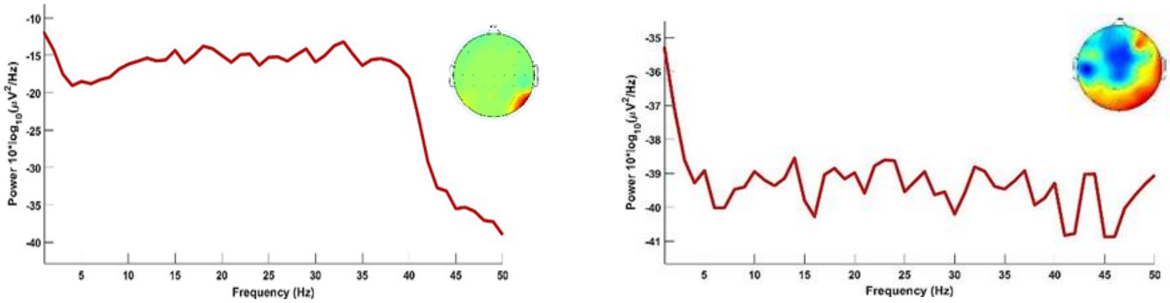

Figure 2. Exemplar activity power spectrum and heat map of brain activity showcasing the 37th (left) and 54th (right) components from participant number 08. These components were excluded due to the absence of a 1/f pattern.

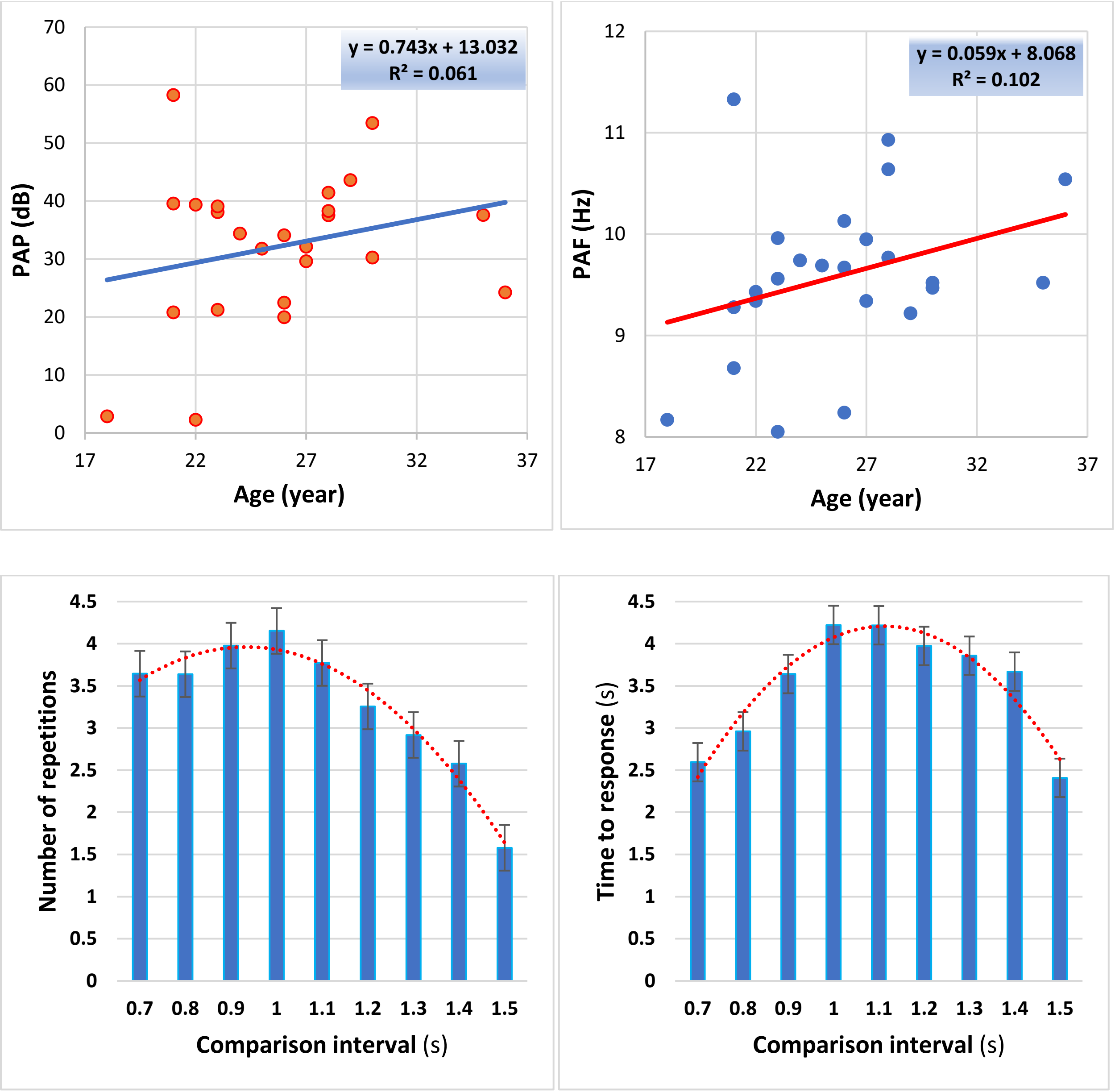

Figure 3. (Top) Age is not a significant predictor for PAP (Left) and PAF (Right). **(Bottom)** Data representations of participants' responses to comparison intervals: (Left) Number of stimulus repetitions needed to respond (μ=3.28±0.27, peak at 1000 ms = 4.15 repetitions, Red fitted curve: $y = -0.07x^2 + 0.48x + 3.16$, $R^2 = 0.97$). (Right) Response time in seconds (μ=3.51±0.23, peak at 1000 ms = 4.22 seconds, Red fitted curve: $y = -0.11x^2 + 1.08x + 1.46$, $R^2 = 0.92$). The data indicates that participants exhibit slower recognition for intervals close to one second.

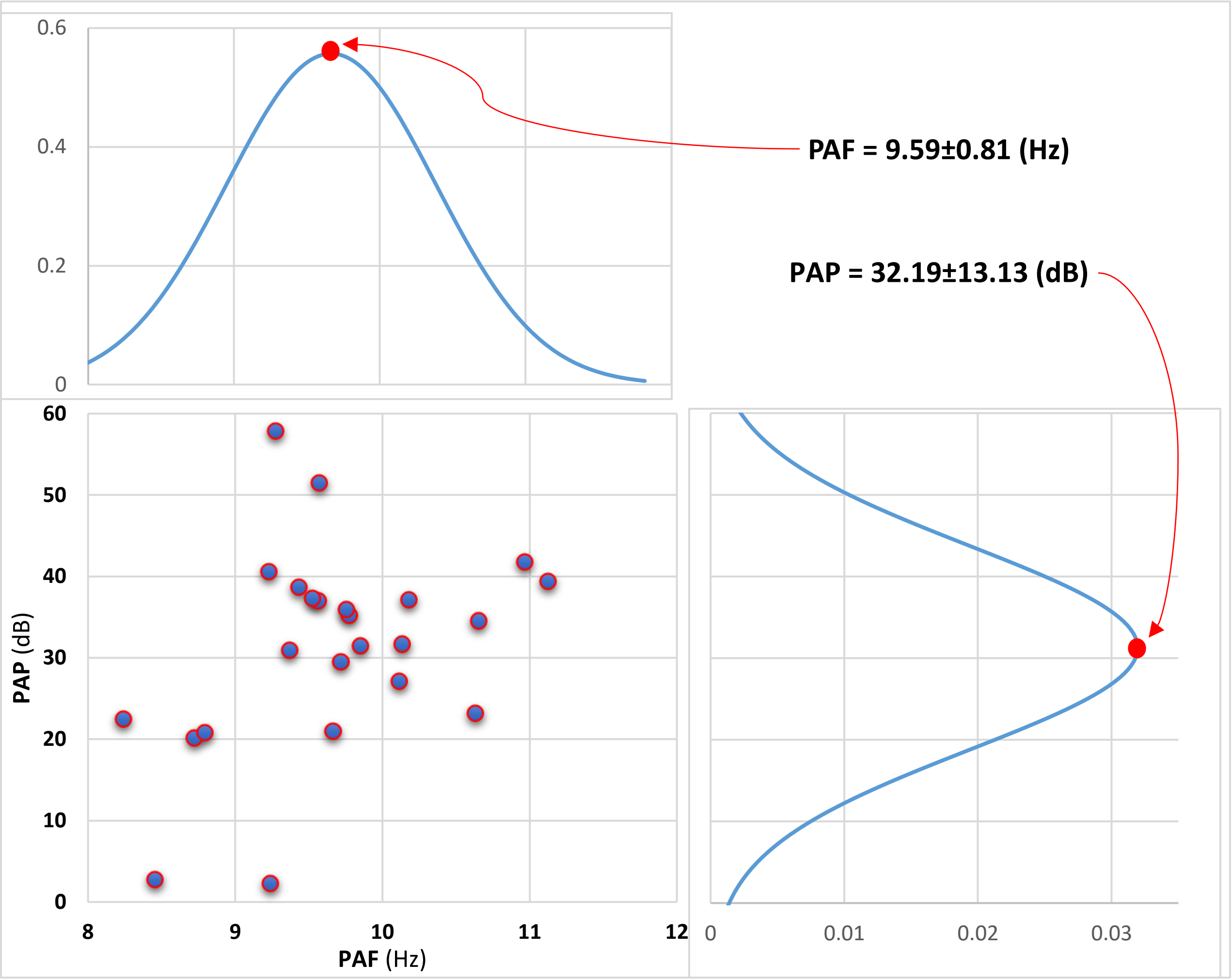

0.6
0.4
0.2
0
PAF = 9.59±0.81 (Hz)
PAP = 32.19±13.13 (dB)
60
50
40
30
20
10
0
PAP (dB)
8
9
10
11
12
PAF (Hz)
0
0.01
0.02
0.03

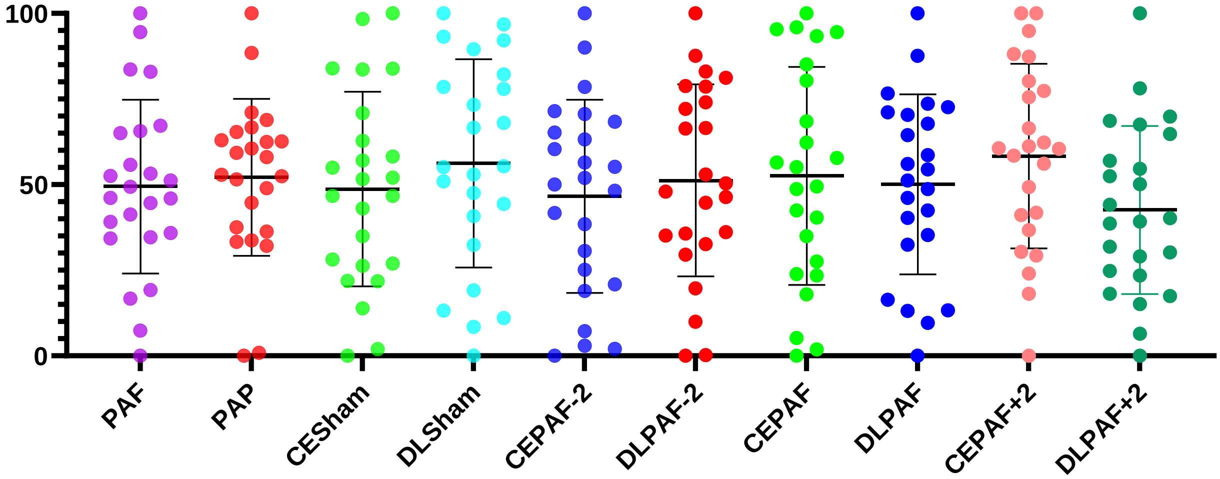

100
50
0
PAF
PAP
CESham
DLSham
CEPAF-2
DLPAF-2
CEPAF
DLPAF
CEPAF+2
DLPAF+2

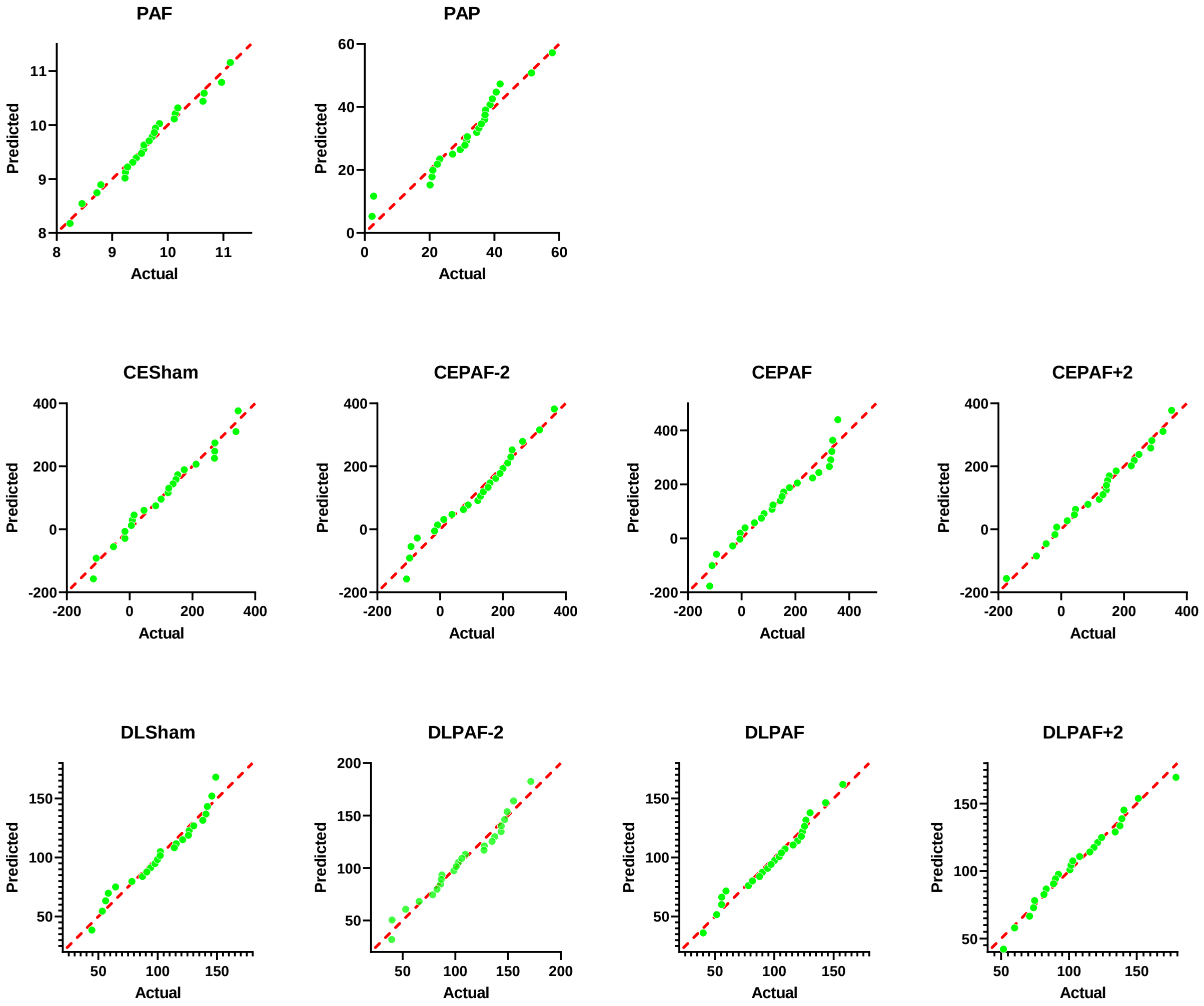

Figure 4. (Top) Scatter plot and normal distribution of PAF and PAP as predictors. Middle: Normal distribution of all parameters. **(Bottom)** Q-Q plots for the parameters.

Table 3. The correlations between predictor and criterion variables for each electrode. It is generally evident that the correlations are more significant for occipital electrodes rather than parieto-occipital ones. Furthermore, correlations are undermined in real stimulations rather than the sham condition. Finally, regarding real stimulations, the correlations become less significant when the stimulation frequency increases. (Dark green cells: p-value < 0.05, Pale green cells: 0.05 < p-value < 0.10)

	Criterion	DL				CE			
Predictor	Stimulation condition / Pearson Correlation	Sham	PAF-2	PAF	PAF+2	Sham	PAF-2	PAF	PAF+2
PAF-O1	Coefficient	-0.420	-0.346	-0.322	-0.317	0.030	0.049	0.085	-0.203
	Sig. (2-tailed)	0.041	0.098	0.125	0.131	0.890	0.820	0.694	0.341
PAF-O2	Coefficient	-0.557	-0.507	-0.467	-0.403	0.089	0.070	0.068	-0.201
	Sig. (2-tailed)	0.005	0.011	0.022	0.051	0.679	0.744	0.752	0.346
PAF-Oz	Coefficient	-0.399	-0.269	-0.262	-0.485	-0.043	0.140	0.071	-0.095
	Sig. (2-tailed)	0.053	0.204	0.216	0.016	0.843	0.513	0.741	0.660
PAF-PO3	Coefficient	-0.478	-0.352	-0.346	-0.313	0.078	0.107	0.126	-0.148
	Sig. (2-tailed)	0.018	0.092	0.097	0.136	0.718	0.618	0.558	0.491
PAF-PO4	Coefficient	-0.345	-0.368	-0.301	-0.202	0.192	0.038	0.144	-0.060
	Sig. (2-tailed)	0.099	0.077	0.154	0.345	0.369	0.862	0.503	0.782
PAF-Poz	Coefficient	-0.413	-0.101	-0.127	-0.318	0.089	0.028	0.155	-0.183
	Sig. (2-tailed)	0.045	0.640	0.555	0.129	0.679	0.898	0.470	0.392
PAP-O1	Coefficient	-0.243	0.037	-0.100	-0.096	0.463	0.417	0.377	0.184
	Sig. (2-tailed)	0.253	0.864	0.640	0.656	0.023	0.043	0.069	0.388
PAP-O2	Coefficient	-0.206	-0.002	-0.099	-0.153	0.509	0.477	0.424	0.213
	Sig. (2-tailed)	0.333	0.994	0.644	0.475	0.011	0.019	0.039	0.317
PAP-Oz	Coefficient	-0.183	0.043	-0.057	-0.116	0.499	0.457	0.420	0.198
	Sig. (2-tailed)	0.391	0.843	0.790	0.589	0.013	0.025	0.041	0.353
PAP-PO3	Coefficient	-0.315	-0.060	-0.223	-0.203	0.375	0.335	0.286	0.107
	Sig. (2-tailed)	0.134	0.781	0.296	0.341	0.071	0.109	0.175	0.617
PAP-PO4	Coefficient	-0.312	-0.075	-0.236	-0.216	0.372	0.321	0.277	0.108
	Sig. (2-tailed)	0.138	0.727	0.268	0.311	0.074	0.126	0.190	0.615
PAP-POz	Coefficient	-0.183	0.042	-0.064	-0.154	0.432	0.384	0.369	0.114
	Sig. (2-tailed)	0.391	0.847	0.768	0.472	0.035	0.064	0.076	0.595
